# Supplementary material for: Identification and Characterization of the Gene Responsible for the O3 Mating Type Substance in Paramecium caudatum
Source: Microorganisms. 2024 Mar 15;12(3):588. doi: 10.3390/microorganisms12030588 (PMC10974281; doi:10.3390/microorganisms12030588)

Figure s1. Multiple amino acid sequence alignment of O^{3}Pc-MSP with 14 homologous proteins. The NCBI accession number for each protein is listed next to the respective species name. Outlined letters denote amino acid residues that are at least 80% identical (dark shade) or chemically similar (light shade). The letters written under the alignment represent highly conserved residues among these proteins.

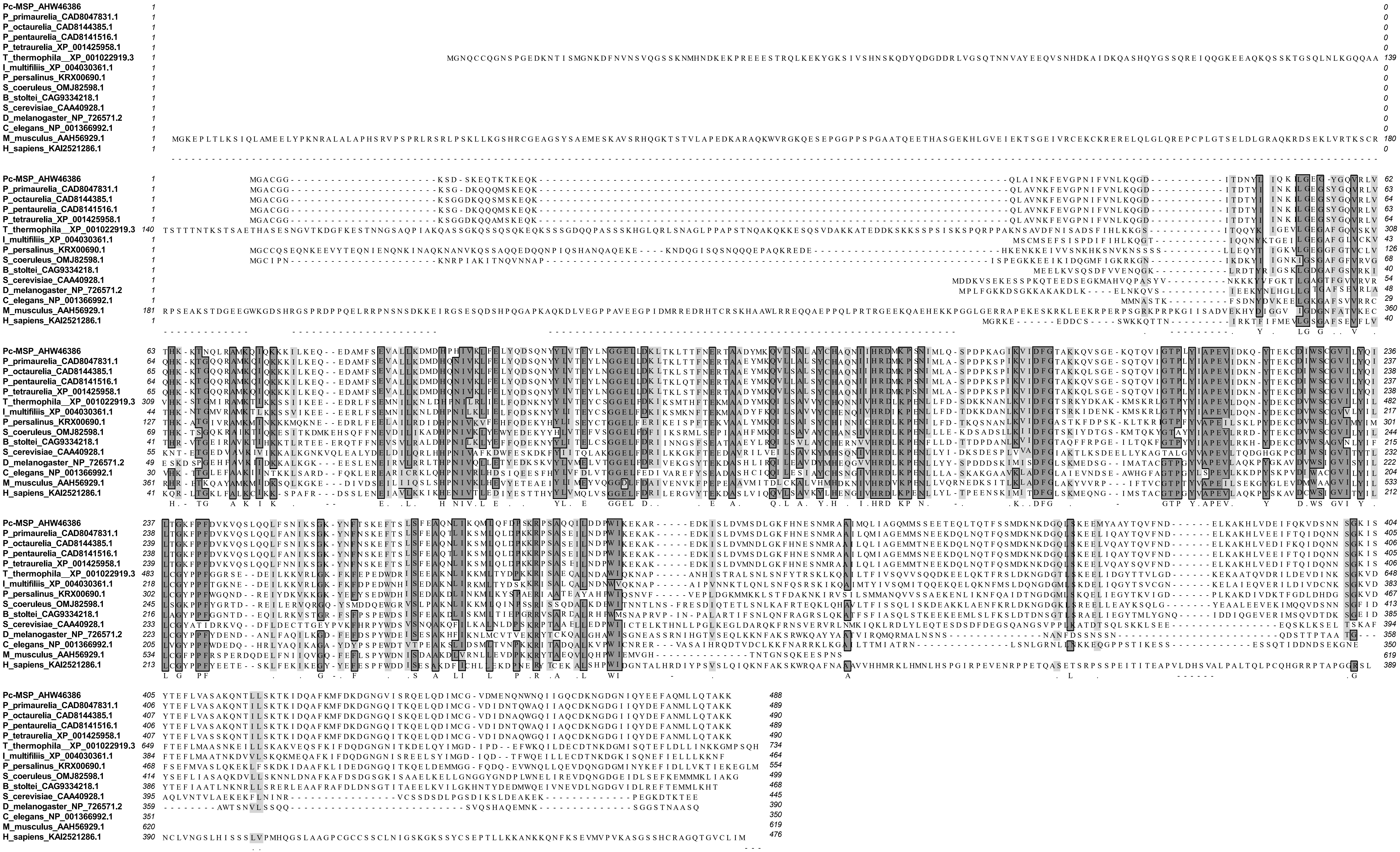

Supplement: Supplementary file 1 [file microorganisms-12-00588-s001.zip › Supplementary Figure S1.pdf]
