# Supplementary material for: Identification and Characterization of the Gene Responsible for the O3 Mating Type Substance in Paramecium caudatum
Source: Microorganisms. 2024 Mar 15;12(3):588. doi: 10.3390/microorganisms12030588 (PMC10974281; doi:10.3390/microorganisms12030588)
Supplement: Supplementary file 1 [file microorganisms-12-00588-s001.zip › Supplymentary Figure S2.pdf]

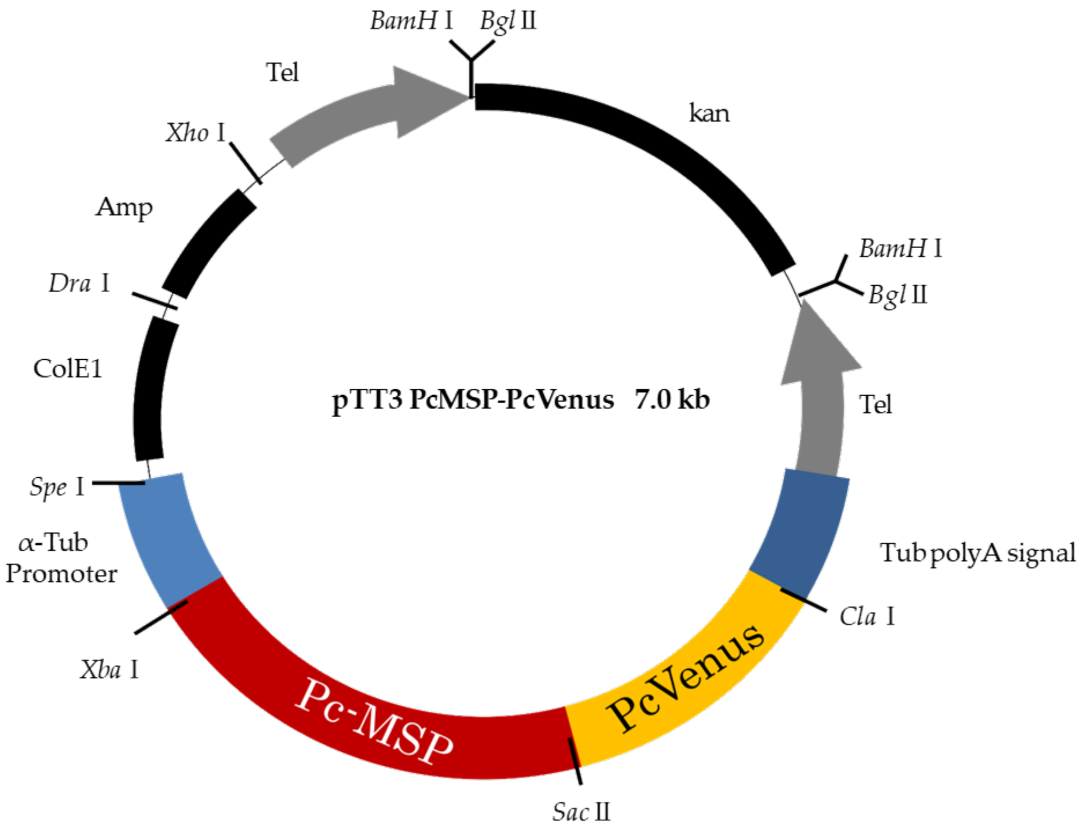

Figure S2: Schematic diagram of pTT3 PcMSP-PcVenus 7.0 kb. The pTT3H2B-pcVenus expression vector was used to create an expression vector carrying the O3PcMSP-PcVenus fusion gene. "Pc-MSP" in the vector diagram refers to O^{3}Pc-MSP. After the vector was cloned and produced in large quantities, it was linearized with the *Bam*HI restriction enzyme and used as a sample for microinjection.
